# First-Principles Investigations of Two-Sided Functionalised MoS_2_ Monolayer

**DOI:** 10.3390/nano15030193

**Published:** 2025-01-26

**Authors:** Sreejita Ray, Beate Paulus

**Affiliations:** Institut für Chemie und Biochemie, Freie Universität Berlin, Arnimallee 22, 14195 Berlin, Germany; rays99@zedat.fu-berlin.de

**Keywords:** transition metal dichalcogenides, molecular functionalisation, alkali metal, F_4_TCNQ, donor-acceptor pairs, first-principles calculations

## Abstract

In this computational study, we investigate two-sided functionalised MoS_2_ with alkali metal atoms as donors and the organic acceptor molecule F_4_TCNQ as an acceptor. Characterisation of functionalised MoS_2_ involves first-principles calculations within the density functional theory (DFT) framework with a PBE+D3 scheme to investigate the electronic structure and quantify the charge transfer in the two-sided functionalised system in comparison to the one-sided functionalised counterpart. Within the two-sided functionalised systems, there is an increase in the overall charge on MoS_2_ as a result of stronger electron transfer from the donor to the monolayer, additionally controlled by the ability of the acceptor to receive electrons.

## 1. Introduction

Over the past two decades, two-dimensional materials (2DMs) have garnered significant attention, owing to the versatile nature of their atomically thin crystalline layers obtained from the exfoliation of the bulk form [1,2,3]. These comprise an entire library of materials which are all beneficial in varied industrial applications, due to their excellent electronic and optical properties and the corresponding flexibility that originates from the modulation of such properties. Graphene is one of the most significant members of this class of compounds, however pristine graphene lacks an electronic band gap which limits its applications in a wide range of optoelectronic devices.

Transition metal dichalcogenides (TMDs) are another member of the family of 2DMs with the chemical formula MX_2_, where M is a transition metal belonging to group IV (Ti, Hf), group V (Nb, Ta) or group VI (Mo, W) and X is a chalcogen (S, Se). Owing to their sizeable electronic band gaps [4,5], TMDs find a fascinating array of applications in optoelectronic devices [6], diodes [7], transistors [8], and semiconductor-integrated circuits [9]. MoS_2_, in particular, undergoes a notable transition from an indirect to a direct electronic bandgap of 1.8 eV when moving from its bulk form to the thermodynamically stable 2H monolayer phase [10].

Research over the past two decades has shown that incorporating molecular functionalities into the system through covalent or non-covalent functionalisation schemes [11,12,13,14,15,16,17] allows for the control of the physicochemical and optoelectronic properties of the 2DMs. The phenomenon of doping, by means of surface adsorption of molecules, especially if they are strong electron donors or acceptors, has a direct impact on the electronic structure of the system as a result of changes in charge carrier density. Previous work in our group has extensively investigated this relationship, exploring the electronic and optical properties of both pristine and defective MoS_2_ as a result of covalent functionalisation and effectively characterised the introduced adsorption states in the fundamental electronic band gap of MoS_2_ [18,19]. The organic molecules 2,3,5,6-tetrafluoro-7,7’,8,8’-tetracyano-quinodimethane (F_4_TCNQ) and 1,3,4,5,7,8-hexafluoro-tetra-cyano-napthoquinodimethane (F_6_TCNNQ) are strong electron acceptors and pertinent examples in this regard. Former research has delved into the diverse adsorption phases involving F_4_TCNQ as a molecular additive on coinage metals to modulate its work function [20,21,22], organic semiconducting polymers [23,24,25,26], graphene [27,28,29,30,31] and other 2DMs [32,33] to consequently influence the total charge carrier density of the pristine materials through controlled doping. The significance of the electronic nature of the substrate in determining the charge transfer mechanisms in van der Waals heterostructures comprising F_6_TCNNQ and monolayer MoS_2_ has been investigated in recent years through both experimental and computational methodologies [34,35]. Consequently, the charge transfer involving the molecular dopant in the heterostructure significantly influences the position of the Fermi level and the sample work function, with additional states induced by p-doping in the TMD. Furthermore, extensive research has been conducted in the past on the role of alkali metals [36,37] as suitable donors in graphite intercalation compounds (GIC) on account of flexible charge transfer from the intercalant to the host material [38,39,40].

In this study, we aim to perform first-principles calculations to investigate the electronic structure of two-sided functionalised MoS_2_ and eventually quantify a possible charge transfer through the MoS_2_ monolayer, from the donor to the acceptor side. For such calculations, the donor atoms of choice are alkali metals while the fluorinated tetracyanoquinone derivative F_4_TCNQ is the acceptor molecule. The choice of alkali metals as donor atoms in our work is guided by the fact that they offer flexibility in controlling the charge transfer to MoS_2_ based on their concentration and coverage per TMD unit cell. In the past, surface adsorption studies involving alkali metals as simplest possible donors on MoS_2_ have been performed, and the extent of preferential binding has been significant in comparison to elements of other groups, however, these do not include the combined effects of an appropriate donor-acceptor pair in the system [41,42]. Similarly, while studies exist on the charge transfer mechanisms involving F_6_TCNNQ on MoS_2_ [34,35], it is particularly interesting to investigate the electronic structure of a model comprising F_4_TCNQ and MoS_2_. This workflow is based on the premise that charge transfer occurs from the donor to the acceptor side through the MoS_2_ monolayer and consequently alters the electronic structure of the the TMD as a result of a two-sided functionalisation scheme.

## 2. Materials and Methods

### 2.1. Computational Details

All spin-polarised DFT calculations were performed with the Vienna ab initio simulation package (VASP) [43]. The interactions between the valence electrons and core ions were described using the projector augmented wave (PAW) formalism [44,45]. Implementing the frozen-core approximation, the electrons in the 4d and 5s orbitals of molybdenum, the 3s and 3p orbitals of sulphur, the 3s orbital of sodium, and the 2s and 2p orbitals of carbon, fluorine, and nitrogen were treated as valence electrons, while the core electrons provided an implicit screening of the ionic potential. A plane-wave basis set associated with kinetic energy values up to 500 eV was chosen. To determine the lattice constant of the MoS_2_ monolayer, structure relaxations were performed with a suitable generalised gradient approximations (GGA)-type functional such as Perdew-Burke-Ernzerhof (PBE) [46] and the Brillouin zone was sampled along a Γ-centred mesh of 12 × 12 × 1. The atomic positions within the systems were allowed to relax until the energy convergence criterion of 10^−5^ eV was achieved, and the norms of all forces acting on the atoms became smaller than 0.01 eV Å^−1^. The blocked-Davidson algorithm was implemented for the electronic minimisation step while ionic relaxations were performed using a conjugate-gradient algorithm, and a Gaussian smearing scheme with a smearing width of 0.05 eV was applied. Furthermore, dispersive interlayer interactions were accounted for using Grimme’s semi-empirical D3 correction scheme [47] along with Becke-Johnson damping [48].

To test the quality of the applied PBE+D3 functional for the electronic band gap, we additionally performed a calculation with the hybrid functional Heyd–Scuseria–Ernzerhof HSE06+D3 [49] for the MoS_2_ monolayer. Whereas for PBE+D3 the band gap was 1.66 eV, it increased to 2.13 eV with the HSE06+D3 treatment. Although the accuracy of the PBE functional with respect to the band gap is not good, its accuracy is sufficient for the study we want to perform. As we have to move to larger supercells to adsorb the acceptor molecule, a PBE+D3 treatment is a sensible balance between accuracy and computing resources.

The total binding energy values $E_{\mathrm{bind}}$ in the one-sided functionalised systems were defined by the following expression:

(1) $$E_{\mathrm{bind}}=E\left({\mathrm{MoS}}_{2}+X\right)-E\left({\mathrm{MoS}}_{2}\right)-nE_{x}$$

where $E({\mathrm{MoS}}_{2}+X)$ denoted the total energy of the one-sided functionalised MoS_2_ monolayer, $E({\mathrm{MoS}}_{2})$ was the energy of pristine MoS_2_ monolayer as an adsorbent and $E_{x}$ was the energy of the isolated adsorbate species with *n* indicating the number of adsorbate groups.

To accurately evaluate the density of states (DOS), the tetrahedron method of smearing with Blöchl corrections was applied, along with a damped velocity friction algorithm for electronic minimisation. The atomic charges in the system were determined using a Bader charge analysis scheme, following the algorithm provided by Henkelman et al. [50].

### 2.2. Model Construction

**Figure 1:**
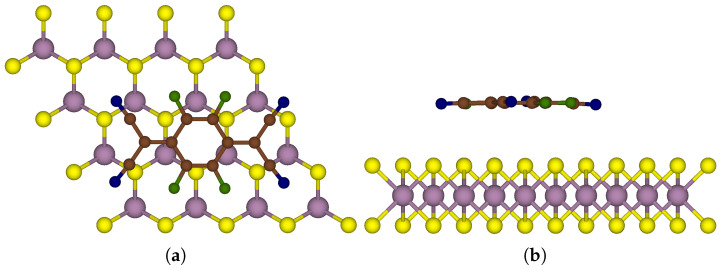
One-sided functionalised MoS_2_ with acceptor molecule F_4_TCNQ on a 4 × 4 supercell; Mo, S, C, F and N atoms are in purple, yellow, brown, green and blue, respectively: (**a**) Top view (**b**) Side view of the F_4_TCNQ-MoS_2_ system.

To investigate surface adsorption, a 4 × 4 supercell model of MoS_2_ was relaxed using a 3 × 3 × 1 K-point sampling. The F_4_TCNQ molecule, in its planar orientation, was placed 2.5 Å away from the 4 × 4 supercell of MoS_2_ as a starting configuration. To prevent interactions within the periodic structure, a vacuum space of 20 Å perpendicular to the MoS_2_ surface was maintained. Figure 1 illustrates the most stable configuration of the F_4_TCNQ molecule adsorbed on MoS_2_, with an associated binding energy of −1.14 eV at a distance of 3.35 Å from the monolayer.

Subsequently, a second model was constructed comprising an F_4_TCNQ molecule with an identical planar orientation to a 6 × 6 supercell of MoS_2_ and subjected to structural relaxations with a 3 × 3 × 1 K-point sampling. The modification in the size of the MoS_2_ supercell resulted in a binding energy of −1.09 eV at a distance of 3.30 Å, therefore, demonstrating no significant impact of the size of the supercell on the characteristics of acceptor binding within the one-sided functionalised system.

## 3. Results and Discussion

### 3.1. One-Sided Functionalisation with Only Donors

**Figure 2:**
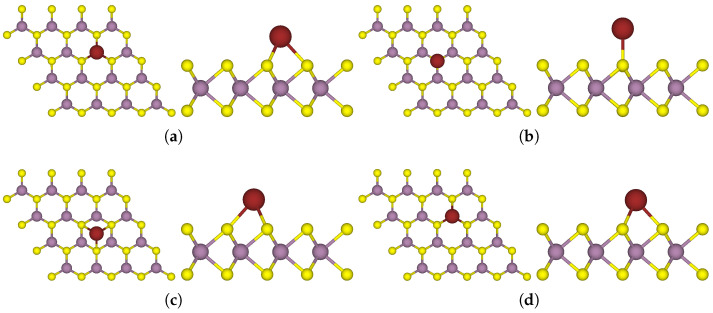
Top and side views of one Na atom at various adsorption sites on a 4 × 4 supercell of MoS_2_. (**a**) Adsorption at the M site which is the hollow site above a Mo atom (**b**) Adsorption at the S site which is the top site above a S atom (**c**) Adsorption at the H site which is the hollow site above the centre of a hexagonal unit in the lattice (**d**) Adsorption at the B site which is the bridge site above halfway of a Mo-S bond.

In this subsection, we aim to explore the influence of alkali metal atoms on the electronic structure of the TMD monolayer. To find the most stable adsorption site of the donor atom, we determined the adsorption energy of a Na atom at various sites on a monolayer of MoS_2_ (for optimised structures, see Figure 2).

**Figure 3:**
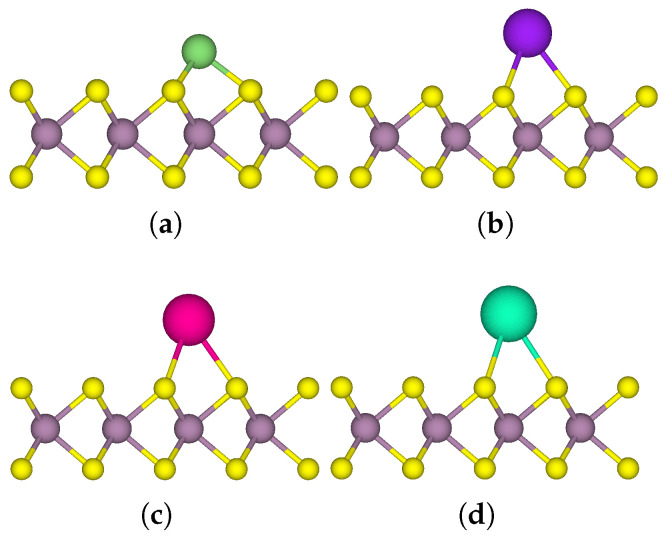
Side views of one-sided functionalised MoS_2_ with different alkali metal atoms adsorbed at the M site on a 4 × 4 supercell of MoS_2_: (**a**) 1Li-MoS_2_ (**b**) 1K-MoS_2_ (**c**) 1Rb-MoS_2_ (**d**) 1Cs-MoS_2_.

The negative binding energy values presented in Table 1 are all greater than 1 eV in magnitude, and therefore, representative of chemisorption. The hollow site above a Mo atom, denoted as the M site, was identified as the most favoured adsorption site. The donor ability of a sodium atom in comparison to other alkali metals, is shown in Figure 3 and Table 1. The charge transfer in the system was quantified by a two-step approach. The systematic Bader partitioning technique of dividing the charge density grid into Bader volumes by zero-flux surfaces, returns values for constituent atomic charges enclosed within these Bader volumes. These atomic charges are then subtracted from the number of valence electrons in the corresponding neutral atoms to yield net atomic charges as listed in Table 1.

**Figure 4:**
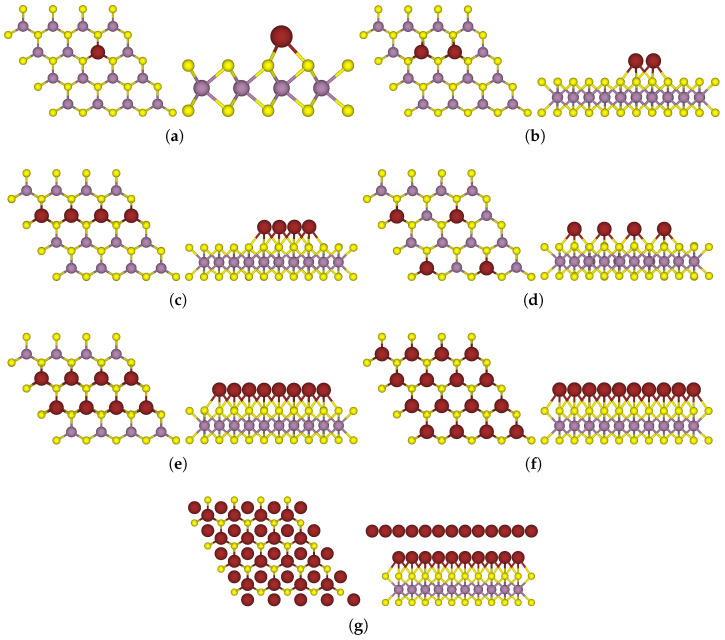
Top and side views of one-sided functionalised MoS_2_ with varying coverage of donor atoms such as 1, 2, 4, 8, 16 and 32 Na atoms (**a**) 1Na-MoS_2_, (**b**) 2Na-MoS_2_, (**c**) 4Na-MoS_2_(a), (**d**) 4Na-MoS_2_(b), (**e**) 8Na-MoS_2_, (**f**) 16Na-MoS_2_, (**g**) 32Na-MoS_2_.

The coverage of the donor atoms was then systematically increased with respect to the MoS_2_ monolayer (Figure 4), and the outcomes are presented in Table 2. While comparing the observations from a Bader charge analysis across different models, it was evident that the system denoted as 4Na-MoS_2_(b), comprising the spaced-out arrangement of four donor atoms on MoS_2_ demonstrated the most substantial average charge transfer per donor atom. In this system, each Na atom donated 0.79 electrons, resulting in a total transfer of 3.16 electrons to the monolayer. As the donor coverage was further increased, there was favourable adsorption indicated by the binding energy per Na atom, however, there was a decrease in the average charge transfer from each donor atom. Furthermore, an additional layer of Na atoms was introduced into the 16Na-MoS_2_ system, resulting in donor atoms being distributed across two layers in both the M and H sites in the 32Na-MoS_2_ system. Concerning the initial layer of Na atoms, the second layer showed a reduced binding affinity of −1.17 eV per Na atom and a nominal average charge transfer of 0.18 electrons per Na atom to MoS_2_. The interlayer distance of 3.65 Å between the Na atoms in 32Na-MoS_2_ is close to the nearest neighbour distance of 3.71 Å for Na metal crystallising in a BCC lattice [51]. This is an indication that the second layer contributed to a minimal charge transfer to MoS_2_ and retained an essentially metallic character. The reduced binding affinity and charge transfer in 32Na-MoS_2_ indicate that the extent of donor functionalisation is reliant on both the electron-donating capacity of the Na atoms and the acceptor characteristics of MoS_2_.

**Figure 5:**
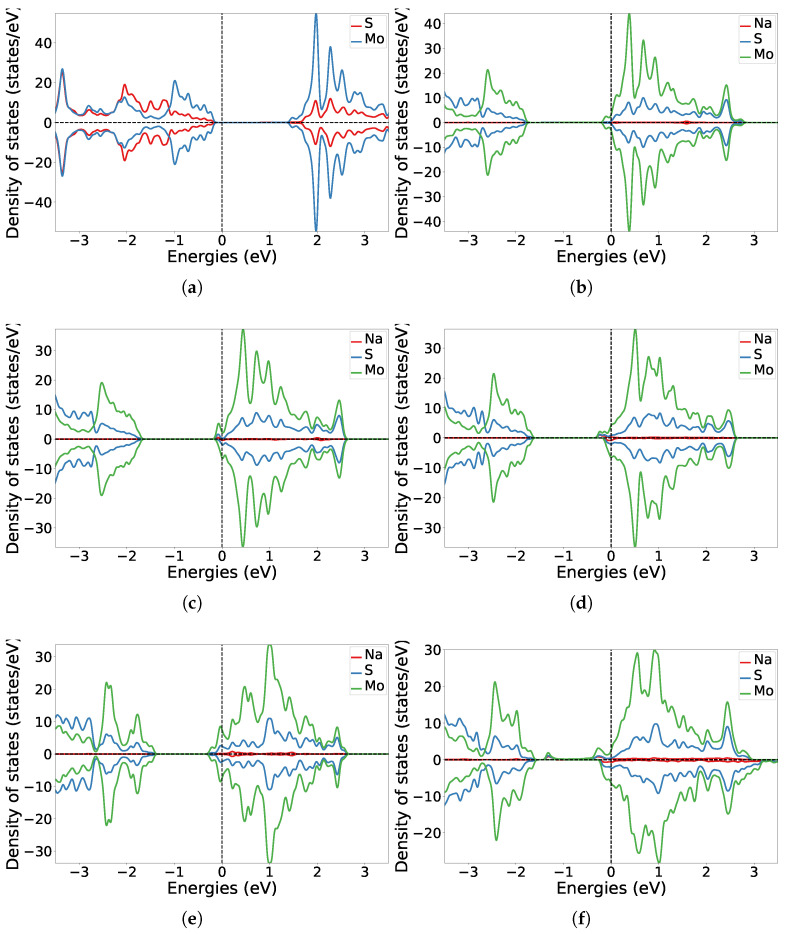

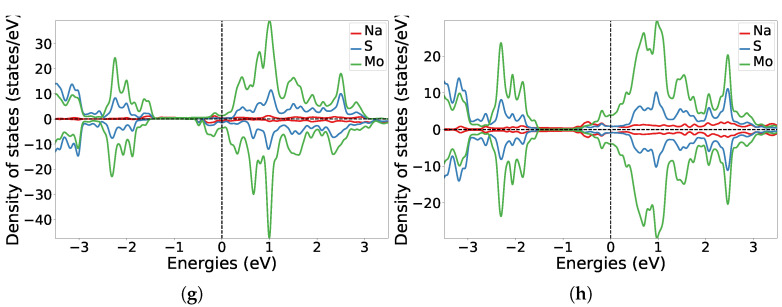
Projected density of states along elements (states/eV) for (**a**) pristine MoS_2_ and varied coverage of donor atoms on MoS_2_: (**b**) 1Na-MoS_2_, (**c**) 2Na-MoS_2_, (**d**) 4Na-MoS_2_(a), (**e**) 4Na-MoS_2_(b), (**f**) 8Na-MoS_2_, (**g**) 16Na-MoS_2_, (**h**) 32Na-MoS_2_. The Fermi energy was positioned at 0 eV in all systems.

For a more comprehensive understanding of the electronic structure of the functionalised systems, the projected DOS along elements is illustrated in Figure 5 and compared to that of pristine MoS_2_. Upon donor functionalisation, the DOS was altered, and there was an upward shift in the Fermi level towards the conduction band of MoS_2_. This was due to the generation of electronic states within the fundamental band gap of the TMD. These donor states, attributed to mobile charge carriers, emerged at the edge of the conduction band as a result of n-doping in the system. Upon the adsorption of a single Na atom, the Fermi level shifted to the bottom of the conduction band with gradual filling of the conduction band as the donor coverage was increased to accommodate up to 32 Na atoms. The intrinsic direct band gap nature of MoS_2_ remained unaffected. However, it was evident that electrons were transferred from the valence 3s orbitals of the Na atom to the 4d orbitals of Mo, making them suitable donors in this case. With a higher coverage of donor atoms as in 8Na-MoS_2_ and 16Na-MoS_2_, the DOS became asymmetric in alpha and beta spins. This was further observed in small magnetic moment values of approximately −0.1 $\mu_{B}$ and −0.01 $\mu_{B}$ on Mo and S respectively, while the values ranged between 0.01 $\mu_{B}$ and 0.05 $\mu_{B}$ for the Na atoms in 16Na-MoS_2_. Additionally, the metallic character of the second layer of the Na atoms in 32Na-MoS_2_ was observed in its corresponding DOS.

### 3.2. Two-Sided Functionalisation with Both Donor and Acceptor

**Figure 6:**
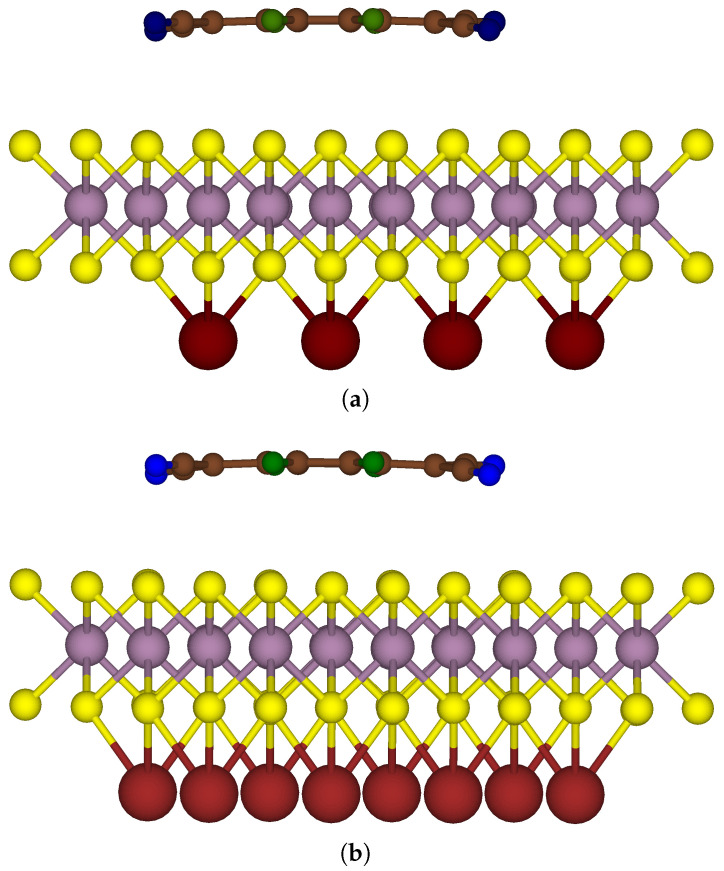
Side view of two-sided functionalised MoS_2_ with an F_4_TCNQ molecule on one side and 4 or 8 Na atoms on the other side: (**a**) F_4_TCNQ-MoS_2_-4Na (**b**) F_4_TCNQ-MoS_2_-8Na.

This section presents an analysis of the characteristics of a model consisting of two-sided functionalised MoS_2_, aiming for a precise comparative evaluation with its one-sided functionalised counterpart. Figure 6 depicts the most stable configuration of a 4 × 4 MoS_2_ supercell with two-sided functionalisation. The system, denoted as F_4_TCNQ-MoS_2_-4Na, in the upper section of Figure 6 comprises the organic acceptor molecule F_4_TCNQ on one side and 4 Na atoms on the other side in their spaced-out arrangement, with the same starting configuration as in the system denoted as 4Na-MoS_2_(b). In the lower section of Figure 6, the extent of donor atom coverage was increased to accommodate 8 Na atoms to build a two-sided functionalised model denoted as F_4_TCNQ-MoS_2_-8Na.

To evaluate the adsorption behaviour in the two-sided functionalised models, the binding energy $E_{\mathrm{bind}}$ was calculated using the following expression:

(2) $$E_{\mathrm{bind}}=E(F_{4}\mathrm{TCNQ-Mo}S_{2}\mathrm{-xNa})-(E(\mathrm{xNa-Mo}S_{2})+E(F_{4}\mathrm{TCNQ}))$$

where $E\left(F_{4}\mathrm{TCNQ-Mo}S_{2}\mathrm{-xNa}\right)$ was the energy of the two-sided functionalised system with x denoting the number of Na atoms, the energy of the one-sided functionalised MoS_2_ with x Na atoms was denoted as $E(\mathrm{xNa-Mo}S_{2})$ and $E\left(F_{4}\mathrm{TCNQ}\right)$ was the energy of the isolated F_4_TCNQ molecule. Thus, the value of $E_{\mathrm{bind}}$ in this case, particularly elucidated the binding affinity of the one-sided donor functionalised MoS_2_ with 4 or 8 Na atoms to the acceptor molecule F_4_TCNQ.

The results listed in Table 3 suggest preferential binding of one-sided donor functionalised MoS_2_ with varying donor coverage to the acceptor molecule. The value of $E_{\mathrm{bind}}$ in the two-sided functionalised MoS_2_ increased to −2.05 eV from −1.14 eV, as in the one-sided acceptor functionalised system, which is further corroborated by the reduced distance between the TMD and the F_4_TCNQ molecule. On the addition of 8 Na atoms in the two-sided functionalised model, the value of $E_{\mathrm{bind}}$ was reduced to −1.77 eV. The acceptor molecule moved slightly closer to MoS_2_ while the nearest Na atom moved farther away to 2.82 Å away from the monolayer in F_4_TCNQ-MoS_2_-8Na, in comparison to 2.69 Å in F_4_TCNQ-MoS_2_-4Na. The electron transfer in the two-sided functionalised MoS_2_ was only slightly higher as compared to the acceptor functionalised MoS_2_ which indicated that the acceptor ability of F_4_TCNQ constrained the extent of charge transfer in the system. The overall charge on the MoS_2_ monolayer in the F_4_TCNQ-MoS_2_ system was 1.07 holes due to the acceptor properties of F_4_TCNQ. With the incorporation of 4 donor atoms as in the two-sided functionalised system, the total charge transferred from the 4 Na atoms amounted to 3.12 electrons, consequently increasing the overall charge on MoS_2_ to 1.90 electrons and switching it from a p-doped to an n-doped material. Furthermore, with an increased coverage of donor atoms as in F_4_TCNQ-MoS_2_-8Na, the charge transfer from each Na atom was reduced to 0.50 electrons, amounting to a total charge transfer of 4 electrons from 8 Na atoms to MoS_2_. Thus, the overall charge on MoS_2_ in this system increased to 2.78 electrons. This confirmed the role of the Na atoms in facilitating additional charge transfer from MoS_2_ to F_4_TCNQ, however, only to a small extent as it is limited by the acceptor ability of F_4_TCNQ.

**Figure 7:**
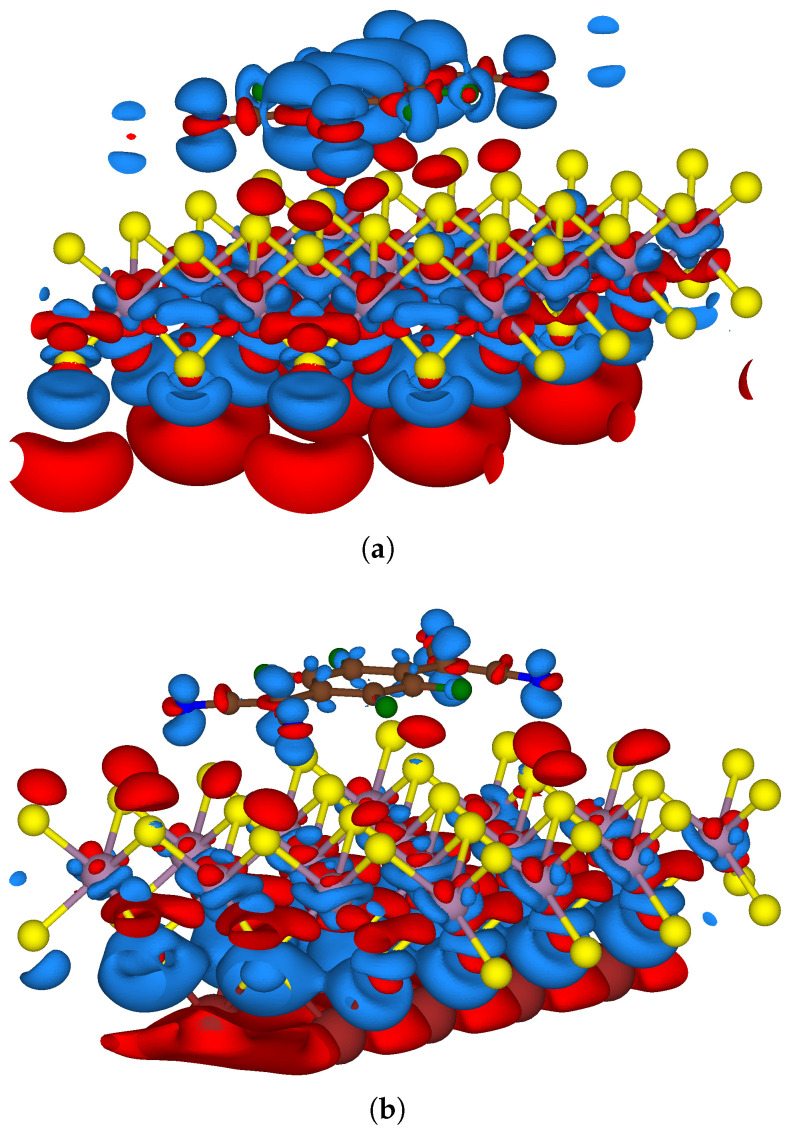
Isosurface plot of charge density difference for two-sided functionalised MoS_2_ in (**a**) F_4_TCNQ-MoS_2_-4Na (**b**) F_4_TCNQ-MoS_2_-8Na; regions of electron density accumulation and depletion are represented in blue and red, respectively. Contour value: 0.001 e/Å^3^.

Figure 7 depicts a contour plot of charge density difference Δρ in the two-sided functionalised systems. The value of Δρ in the two-sided functionalised systems was calculated with the following approach:

(3) $$\Delta \rho =\rho \left(F_{4}\mathrm{TCNQ-Mo}S_{2}\mathrm{-xNa}\right)-(\rho \left(\mathrm{Mo}S_{2}\right)+\rho \left(\mathrm{xNa}\right)+\rho \left(F_{4}\mathrm{TCNQ}\right))$$

where $\rho \left(F_{4}\mathrm{TCNQ-Mo}S_{2}\mathrm{-xNa}\right)$ referred to the charge density of the two-sided functionalised systems, $\rho \left(\mathrm{Mo}S_{2}\right)$ was the charge density of pristine MoS_2_, $\rho \left(\mathrm{xNa}\right)$ and $\rho \left(F_{4}\mathrm{TCNQ}\right)$ referred to that of the individual donor and acceptor components, respectively with x denoting 4 or 8 Na atoms.

This diagram illustrates a region of pronounced charge depletion around the Na atoms and a charge accumulation over the F_4_TCNQ molecule with a dependence of Δρ on the coverage and arrangement of sodium atoms on MoS_2_. In F_4_TCNQ-MoS_2_-4Na, the charge transfer with respect to the acceptor molecule was located more uniformly at the centre of the monolayer while in F_4_TCNQ-MoS_2_-8Na the charge was transferred across different regions of MoS_2_. Thus, the S atoms in the monolayer proximal to F_4_TCNQ had different charge densities in both of the two-sided functionalised models which led to an overall larger positive charge on MoS_2_ in F_4_TCNQ-MoS_2_-8Na. Furthermore, in the presence of 4 Na atoms, a larger extent of negative charge transfer to the π-system and positive charge transfer to the σ-system in the acceptor molecule was observed, in comparison to the system with 8 Na atoms. This indicated that the different coverage and arrangement of the Na atoms is able to influence both the extent and position of charge transfer between MoS_2_ and F_4_TCNQ. Further investigations, including a detailed analysis of the occupied and unoccupied orbitals of isolated F_4_TCNQ and their changes induced by adsorption, will be necessary to elucidate the intricate interplay between donor coverage and arrangement and the resultant charge distribution within the acceptor molecule.

**Figure 8:**
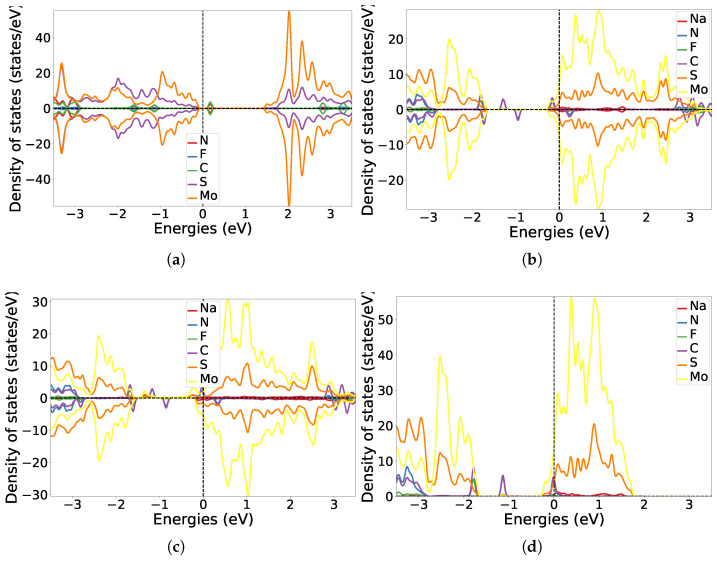
Projected density of states along elements (states/eV) for one-sided and two-sided functionalised MoS_2_: (**a**) F_4_TCNQ-MoS_2_, (**b**) F_4_TCNQ-MoS_2_-4Na, (**c**) F_4_TCNQ-MoS_2_-8Na, (**d**) F_4_TCNQ-MoS_2_-4Na (spin restricted). The Fermi energy was positioned at 0 eV in all systems.

Figure 8 illustrates the projected DOS in the one-sided and two-sided functionalised systems. As a result of electron transfer from MoS_2_ to F_4_TCNQ, additional acceptor states belonging to the C atom appeared within the intrinsic band gap of the system above the valence band edge. This further corroborated electrons being transferred from MoS_2_ to the unoccupied orbitals of F_4_TCNQ. In the two-sided functionalised systems, additional donor states appeared at the lower edge of the conduction band due to the presence of the Na atoms in the systems. With up to 8 Na atoms in the system, more donor states were introduced into the electronic band gap of MoS_2_ and the Fermi energy shifted further up to the conduction band by about 0.2 eV. This demonstrated the change in electronic properties of the TMD monolayer on functionalisation, since MoS_2_ was p-doped in the acceptor functionalised system and n-doped on two-sided functionalisation. The DOS in the two-sided functionalised systems displayed an antisymmetry in states belonging to the C atom. To explain this behaviour, we performed spin-restricted DFT calculations on F_4_TCNQ-MoS_2_-4Na and the total energy in this case was higher by 50 meV than that of the spin-unrestricted calculations (Figure 8d). Furthermore, the spin-projected DOS in F_4_TCNQ-MoS_2_-4Na was visualised to indicate a more prominent α-spin character on F_4_TCNQ as compared to the β-spin. The antisymmetric characteristic of the DOS for both of the two-sided functionalised systems was represented by varying values of positive and negative magnetic moments on the C atoms in the acceptor molecule. Finite values of magnetic moments on F_4_TCNQ were observed in these systems, however, further investigation is required to characterise these effects.

## 4. Conclusions

Our first-principles calculations indicated a reasonably large extent of charge transfer to both one-sided and two-sided functionalised MoS_2_ on account of n-doping due to donor adsorption. This was investigated by increasing the coverage of Na atoms on the MoS_2_ monolayer and comparing the preferential binding in each system. The charge transfer in such one-sided donor functionalised systems was dependent on both donor coverage and the electron accepting properties of MoS_2_. Our study was then extended to two-sided functionalised MoS_2_ with F_4_TCNQ as acceptor and two differing coverages of donor atoms in the systems. The overall charge transfer to the acceptor molecule increased from 1.07 holes to 1.22 holes, with the addition of Na atoms in the system. However, this was limited by the acceptor capacity of F_4_TCNQ, as an increased incorporation of Na atoms elevated the electronic charge within the MoS_2_ monolayer without affecting the charge on the acceptor. These investigations additionally demonstrated that the charge density on F_4_TCNQ could be modulated by varying the coverage and arrangement of donor atoms in the two-sided functionalised systems. To summarise, the overall charge on the MoS_2_ monolayer was increased as a result of stronger electron transfer from the donor to the acceptor side within a two-sided functionalisation scheme.

## Figures and Tables

**Table 1:** Calculated properties of one-sided functionalised MoS_2_ with different alkali metal atoms: adsorption site on MoS_2_, total binding energy (E_bind_) in eV, distance between the alkali metal atom and the MoS_2_ monolayer (d_(metal-ML)_) in Å and charge transfer occurring from the alkali metal to MoS_2_ in electrons.

System	Adsorption Site	E_bind_ (eV)	d_(metal-ML)_ (Å)	Charge Transfer (e)
1Na-MoS_2_	M	−1.46	2.73	0.85
1Na-MoS_2_	S	−1.05	2.60	0.79
1Na-MoS_2_	H	−1.44	2.74	0.84
1Na-MoS_2_	B	−1.46	2.74	0.84
1Li-MoS_2_	M	−1.97	2.35	0.87
1K-MoS_2_	M	−1.84	3.08	0.89
1Rb-MoS_2_	M	−1.87	3.23	0.90
1Cs-MoS_2_	M	−1.90	3.40	0.90

**Table 2:** Calculated properties of one-sided functionalised MoS_2_ with a varied coverage of donor atoms: total binding energy (E_bind_) in eV, the binding energy per Na atom (E_bind_/Na) in eV, the average distance between the Na atom and the MoS_2_ monolayer (d_(Na-ML)_) in Å and average charge transfer occurring from each Na atom to MoS_2_ in electrons.

System	E_bind_ (eV)	E_bind_/Na (eV)	d_(Na-ML)_ (Å)	Charge Transfer (e)
1Na-MoS_2_	−1.46	−1.46	2.73	0.85
2Na-MoS_2_	−2.62	−1.31	2.74	0.67
4Na-MoS_2_(a) *	−4.60	−1.15	2.80	0.51
4Na-MoS_2_(b) ^†^	−4.92	−1.23	2.67	0.79
8Na-MoS_2_	−10.24	−1.28	2.82	0.46
16Na-MoS_2_	−20.64	−1.29	2.93	0.36
32Na-MoS_2_	−39.36	−1.23	2.88	0.18

* The average distance between two Na atoms is 3.16 Å; ^†^ The average distance between two Na atoms is 6.36 Å.

**Table 3:** Calculated properties of two-sided functionalised MoS_2_: binding energy (E_bind_) in eV, the average distance between the F_4_TCNQ molecule and the MoS_2_ monolayer (d_(F_4_TCNQ-ML)_) in Å and the average charge transfer occurring from F_4_TCNQ in electrons.

System	E_bind_ (eV)	d_(F_4_TCNQ-ML)_ (Å)	Charge Transfer (e)
F_4_TCNQ-MoS_2_	−1.14	3.35	−1.07
F_4_TCNQ-MoS_2_-4Na	−2.05	3.24	−1.22
F_4_TCNQ-MoS_2_-8Na	−1.77	3.21	−1.22

## Data Availability

The data is contained within the article.

## Abbreviations

The following abbreviations are used in this manuscript:

TMD	transition metal dichalcogenide
2DM	two-dimensional material
GIC	graphite intercalation compound
DOS	density of states
DFT	density functional theory
GGA	generalised gradient approximations
PBE	Perdew-Burke-Ernzerhof
HSE	Heyd–Scuseria–Ernzerhof
